# Hepatoprotective and Antioxidant Effect of *Bauhinia hookeri* Extract against Carbon Tetrachloride-Induced Hepatotoxicity in Mice and Characterization of Its Bioactive Compounds by HPLC-PDA-ESI-MS/MS

**DOI:** 10.1155/2014/245171

**Published:** 2014-05-14

**Authors:** Eman Al-Sayed, Olli Martiskainen, Sayed H. Seif el-Din, Abdel-Nasser A. Sabra, Olfat A. Hammam, Naglaa M. El-Lakkany, Mohamed M. Abdel-Daim

**Affiliations:** ^1^Department of Pharmacognosy, Faculty of Pharmacy, Ain-Shams University, Cairo 11566, Egypt; ^2^Laboratory of Organic Chemistry and Chemical Biology, Department of Chemistry, University of Turku, 20014 Turku, Finland; ^3^Department of Pharmacology, Theodor Bilharz Research Institute, Giza 12411, Egypt; ^4^Department of Pathology, School of Medicine, Stanford University, Stanford, CA 94305, USA; ^5^Department of Pharmacology, Faculty of Veterinary Medicine, Suez Canal University, Ismailia 41522, Egypt; ^6^Center for Emerging Infectious Diseases, Gifu University, 1-1 Yanagido, Gifu 501-1194, Japan

## Abstract

The hepatoprotective and antioxidant activity of *Bauhinia hookeri* ethanol extract (BHE) against CCl_4_-induced liver injury was investigated in mice. BHE was administered (500 and 1000 mg/kg/day) along with CCl_4_ for 6 weeks. The hepatic marker enzymes: alanine aminotransferase (ALT), aspartate aminotransferase (AST), and alkaline phosphatase (ALP) were determined in the serum. The antioxidant parameters: glutathione (GSH), superoxide dismutase (SOD), glutathione peroxidase (GPx), glutathione reductase (GR), glutathione transferase (GST), and malondialdehyde (MDA) were estimated in the liver homogenate. BHE treatment significantly inhibited the CCl_4_-induced increase in ALT (44 and 64%), AST (36 and 46%), ALP (28 and 42%), and MDA (39 and 51%) levels at the tested doses, respectively. Moreover, BHE treatment markedly increased the activity of antioxidant parameters GSH, GPx, GR, GST, and SOD. Histological observations confirmed the strong hepatoprotective activity. These results suggest that a dietary supplement of BHE could exert a beneficial effect against oxidative stress and various liver diseases by enhancing the antioxidant defense status, reducing lipid peroxidation, and protecting against the pathological changes of the liver. The hepatoprotective activity of BHE is mediated, at least in part, by the antioxidant effect of its constituents. The active constituents of BHE were identified by HPLC-PDA-ESI/MS/MS.

## 1. Introduction

The liver is a vital organ that has a crucial role in the detoxification of various xenobiotics [1]. Excessive exposure to drugs and environmental pollutants overpowers the natural protective mechanisms of the liver and leads to hepatic injury. Liver damage is associated with cellular necrosis, plasma membrane damage, and depletion in the glutathione level (GSH). The levels of serum markers of liver damage such as alanine aminotransferase (ALT), aspartate aminotransferase (AST), and alkaline phosphatase (ALP) are elevated [2]. Long-standing hepatic injury leads to hepatic steatosis, fibrosis, and even to life-threatening conditions, such as liver cirrhosis and hepatocellular carcinoma [3]. Steroids and vaccines have been used for the treatment of liver diseases; however, they have serious adverse side effects and are of limited therapeutic benefits [3]. Silymarin, a mixture of flavonolignans obtained from milk thistle, is a popular herbal extract used as a hepatoprotective agent [4]. However, some clinical trials have indicated that the standard doses of silymarin were ineffective in many patients with chronic liver disease [5]. In view of the limited therapeutic options available for the treatment of liver diseases, the search for new and safe hepatoprotective candidates is quite apparent.

Herbal medicines have been used for centuries for the treatment of several ailments. Natural products remain as important sources of lead structures for the development of many drugs [4]. Recently, there has been a resurgence of interest in the use of natural products because of their reduced side effects when compared to synthetic drugs [3]. Plants are considered as potential hepatoprotective agents because they contain a combination of different phytochemicals that are synergistic in their action [6, 7]. Dietary polyphenolic compounds are abundant in many plant foods such as fruits, vegetables, and legumes. There is growing interest in the use of dietary polyphenols because of their beneficial health effects [8]. Dietary polyphenolic compounds help to restore the balance between the natural antioxidants and free radicals by enhancing the activity of natural antioxidant defenses such as superoxide dismutase (SOD), glutathione peroxidase (GPx), glutathione reductase (GR), and glutathione-*S*-transferase (GST) and by direct scavenging of free radicals [8, 9].

The genus* Bauhinia* (Fabaceae) comprises 300 species and are commonly known as “cow's paw” tree, because of the shape of their leaves [10]. Plants of the genus* Bauhinia* are widely distributed in Africa, Asia, and South America. The leaves and stem-bark of these plants have been used in folk medicine for the treatment of different ailments [10]. Several pharmacological activities have been reported for many* Bauhinia* species, including hepatoprotective, antioxidant, anti-inflammatory, and antihyperlipidemic effects [11, 12].* B. hookeri* F. Muell is a small ornamental tree native to Australia [13]. Although various pharmacological effects were reported for different* Bauhinia* species, no studies have so far been conducted on the chemical constituents and the pharmacological activities of* B. hookeri.* The first objective of this study was to determine the antioxidant and hepatoprotective effect of the phenolic-rich ethanol extract of* B. hookeri* (BHE) against CCl_4_-induced hepatotoxicity in mice, with the aim to develop a safe and effective hepatoprotective agent. Hepatoprotection was determined by assaying the activities of ALT, AST, and ALP in the serum of control and treated mice. The lipid peroxidation and antioxidant parameters (SOD, GSH, GPx, GR, and GST) were estimated in the liver homogenates to determine the possible mechanisms of the hepatoprotective activity. A histopathological examination of liver sections was conducted to confirm the hepatoprotective effect. The second objective of the present study was to identify the bioactive constituents of* B. hookeri*, utilizing the HPLC-PDA-ESI/MS/MS technique (high-performance liquid chromatography coupled with diode array detection-electrospray ionization mass spectrometry).

## 2. Materials and Methods

### 2.1. Chemicals

Carbon tetrachloride (CCl_4_), ethylenediaminetetraacetic acid (EDTA), hydrogen peroxide (H_2_O_2_), 5,5′-dithiobis-2-nitrobenzoic acid (DTNB, Ellman's reagent), potassium dihydrogen phosphate (KH_2_PO_4_), reduced glutathione (GSH), 1-chloro-2,4-dinitrobenzene (CDNB), nicotinamide adenine dinucleotide phosphate reduced form (NADPH), nicotinamide adenine dinucleotide reduced form (NADH), GR, oxidized glutathione (GSSG), nitroblue tetrazolium (NBT), phenazine methosulphate (PMT), trichloroacetic acid (TCA), thiobarbituric acid (TBA), and silymarin were purchased from Sigma-Aldrich Chemical Co. (St. Louis, MO, USA). The assay kits were purchased from Spectrum, MDSS, GmbH, Hannover, Germany.

### 2.2. Plant Material

The leaves of* B. hookeri* were collected in July 2011 from the botanical garden of the Faculty of Agriculture, Cairo University, Cairo, Egypt. The plant was botanically identified by Eng. Therese Labib, the taxonomy specialist at the herbarium of El-Orman Botanical Garden, Giza, Egypt. A voucher specimen of* B. hookeri* was deposited at the herbarium of the Department of Pharmacognosy, Faculty of Pharmacy, Ain Shams University, Cairo, Egypt (ASU BHF2011).

### 2.3. Extract Preparation

Air-dried powdered leaves of* B. hookeri* (1700 g) were extracted three times with 80% EtOH. The total extract was concentrated and freeze-dried to obtain a dry powder, which was dissolved in absolute EtOH. The EtOH-soluble portion was concentrated and freeze-dried to obtain a dry powder of BHE (140 g).

### 2.4. HPLC-PDA-ESI/MS/MS Method

Part of the extract was dissolved in 20% MeOH (20 mg/mL), and the solution was filtered through 0.45 *μ*m membranes. LC-HRESIMS was performed on a Bruker micrOTOF-Q Daltonics (API) Time-of-Flight mass spectrometer (Bremen, Germany) coupled to a 1200 series HPLC system (Agilent Technologies, Waldbronn, Germany) equipped with Starlight diode-array detector (PDA). Chromatographic separation was performed on an XBridge C18 (2.1 × 100 mm; 3.5 *μ*m) column (Waters, Dublin, Ireland). The mobile phase consisted of acetonitrile (A) and 0.1% formic acid (B). The elution profile was 0–3 min, 100% B (isocratic); 3–30 min, 0–30% A in B; 30–35 min, 30–70% A in B; 35–37 min, 70% A in B (isocratic) with constant flow rate of 0.2 mL/min. The HPLC system was controlled by Hystar software (version 3.2; Bruker BioSpin GmbH, Rheinstetten, Germany). The mass spectrometer was controlled by the Compass 1.3 for micrOTOF software package (Bruker Daltonics GmbH, Rheinstetten, Germany). The ionization technique was a pneumatically assisted electrospray. The mass spectrometer was operated in the negative mode, and mass detection was performed in the full scan mode in the range of* m/z* 50–2000* m/z*. The following settings were applied to the instrument: capillary voltage, 4000 V; end plate offset −500 V. The drying gas (N_2_) flow rate was 8.4 L/min, and the drying gas temperature was 200°C. For collision-induced dissociation (CID) MS/MS measurements, the voltage over the collision cell varied from 20 to 70 eV. Argon was used as collision gas. The data were analyzed using Compass Data Analysis Software (version 4.0 SP5; Bruker Daltonics GmbH, Rheinstetten, Germany).

### 2.5. Animals

Male Swiss albino mice (CD-1 strain) weighing 20–25 g were purchased from the Schistosome Biological Supply Center at the Theodor Bilharz Research Institute, Giza, Egypt. The animals were housed under standard laboratory conditions of controlled temperature (20 ± 2°C), humidity (60 ± 5%), and under 12 h light and dark cycles. The mice were fed a standard rodent pellet chow and water* ad libitum*. The animals were acclimatized for at least l week before use. All the animal experiments were conducted in accordance with the Guide for the Care and Use of Laboratory Animals of the National Institutes of Health (NIH 1985) and were approved by the ethical committee of the Faculty of Pharmacy, Ain Shams University, Cairo, Egypt (ASU 2013-8 Research Article 5, approval date August 5th 2013).

### 2.6. Acute Toxicity Study

A group of 36 normal male mice weighing 20–25 g were used to determine the acute oral toxicity of BHE according to the previous method [14]. The mice were divided into six subgroups (*n* = 6 per group). The subgroups were treated with graded doses (500, 1000, 2000, 3000, 4000, and 5000 mg/kg body weight p.o.) of BHE. The animals were observed for 24 h to record toxicity symptoms and mortality rates.

### 2.7. Animal Grouping and Experimentation

The animals were divided into five groups (*n* = 9 per group). Group (I) served as the normal control and received vehicle only (0.5%, w/v, carboxymethyl cellulose). Group (II) was treated intraperitoneally with a sublethal dose of CCl_4_ (20% CCl_4_/olive oil, 3 days/week) for 6 weeks to induce chronic liver injury. The mice in groups (III and IV) were treated orally with 500 and 1000 mg/kg body weight of BHE, respectively, together with CCl_4 _for 6 weeks. The extract was given to the animals 5 days/week, while CCl_4_ was administered 3 days/week. Group (V) was treated with standard silymarin at a daily dose of 500 mg/kg body weight p.o., 5 days/week. Similarly, CCl_4_ was administered 3 days/week. The animals were anesthetized using diethyl ether and then sacrificed by decapitation 48 h after the last treatment dose and blood samples were immediately collected. The livers were dissected to two parts. The first part was fixed in 10% formalin for the histopathology. The second part was washed with 0.9% ice cold saline. A piece of 0.5 g was homogenized in 2.5 volumes (w/v) ice cold 0.1 M potassium phosphate buffer (pH 7.4). The homogenate was centrifuged at 600* g* for 10 min to remove the cell debris, and the supernatant was centrifuged at 10000* g* and the pellet was discarded. The supernatant was collected and stored at −70°C for the estimation of liver GSH content, antioxidant enzymes, and lipid peroxidation.

### 2.8. Biochemical Assays

The collected blood was allowed to clot, the serum was separated at 1800* g*, and the biochemical markers of hepatic damage, including serum AST (U/L), ALT (U/L), and ALP (IU/L), were estimated according to previously reported methods [15, 16]. Liver homogenate was deproteinized in 5% (w/v) TCA, centrifuged at 2000* g* for 20 min, and the GSH content was estimated by Ellman's reagent using a standard curve [17]. The SOD assay was based on the inhibition of NBT reduction to water insoluble blue formazan [18]. One unit of enzyme activity was defined as the amount of enzyme that causes half-maximal inhibition of NBT reduction in the NADH-PMT-NBT reaction system. The SOD activity was assayed at 560 nm and expressed in *μ*mole/min/g liver. The hepatic GPx activity was determined by monitoring GSH oxidation [19]. The enzyme reaction contained NADPH, GSH, and GR and was initiated by the addition of H_2_O_2_. The change in absorbance was monitored at 340 nm. One unit of GPx activity was defined as the amount of enzyme that catalyzes the oxidation of 1 *μ*mole of NADPH/min/g liver. The GR activity was assayed by monitoring the oxidation of NADPH at 340 nm using GSSG as a substrate [20]. The GST activity was measured based on the rate of increase in the conjugate formation between GSH and CDNB, and the absorbance was monitored at 340 nm. One unit of GST activity was defined as 1 *μ*mole conjugate formation/min/g liver [21]. Malondialdehyde (MDA) is a biomarker of lipid peroxidation and reacts with TBA to form a pink chromogen [22]. Briefly, 1 mL of the liver homogenate was mixed with 1 mL of TCA (10% w/v) and was centrifuged at 1850* g* for 15 min. 1 mL of TBA solution (0.67% w/v) was added to 1 mL of supernatant and boiled for 45 min. Absorbance was read after cooling at 530 nm against a blank containing all the reagents except the liver homogenate. The content of thiobarbituric acid reactive substances (TBARS) in the samples was calculated using the extinction coefficient of MDA (1.56 × 10^5^ M^−1 ^cm^−1^), and the results were expressed as MDA equivalents in nmole/g liver.

### 2.9. Histopathological Examination

Liver sections were embedded in paraffin and sliced into 5 *μ*m thick sections in a rotary microtome (Leica, USA) and then stained with hematoxylin-eosin dye (Merck). The histopathological examination of the slides was performed under a microscope (Zeiss, Germany) with ×200 magnification power. The parameters examined for the assessment of histological damage and their relative score systems were as follows:

1. hepatic architecture: preserved, partial loss or complete loss,
2. hydropic degeneration: absent, mild, moderate or marked,
3. fatty changes: absent or present,
4. central vein congestion: present or absent,
5. Kupffer cell hyperplasia: absent or present,
6. necrosis: absent or present,
7. infiltration of portal tract by lymphocytes: absent or present.

### 2.10. Statistical Analysis

All the data were expressed as means ± SEM. Statistical analysis of the data was performed using the one-way ANOVA test followed by Tukey's* post hoc* test to determine the difference between the mean values of the different groups. All statistical analyses were performed using the GraphPad InStat software (Version 3.06, La Jolla, CA, USA). *P* values < 0.05 were considered statistically significant.

## 3. Results

### 3.1. Identification of the Constituents of BHE by HPLC-PDA-ESI/MS/MS

**Figure 1:**
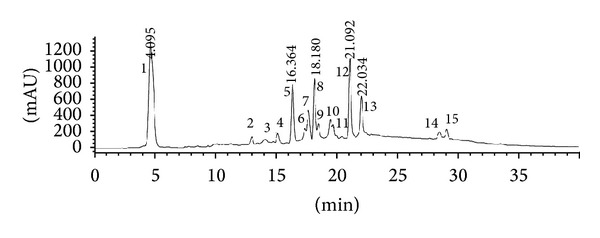
HPLC chromatogram of BHE.

In this study, 15 compounds were detected in BHE using the HPLC instrument coupled to a PDA detector and a mass spectrometer. The combination of the PDA and mass spectrometry (MS/MS) data provided a sensitive method for the characterization of the constituents of BHE (Table 1 and Figure 1). Compounds 2 and 3 were identified as caffeic pentose esters based on their molecular ion [M-H]^−^ at* m*/*z* 311.04 and the MS/MS ion at* m*/*z* 179.04 of caffeic acid, which results from the loss of a pentose moiety (−132 amu). A base peak at* m*/*z* 135.05 was detected corresponding to a typical loss of a carboxylic group from the caffeic acid unit [23]. Compound 5 showed a molecular ion [M-H]^−^ at* m*/*z* 295.05. The loss of a pentose unit resulted in diagnostic peaks of coumaric acid at* m*/*z* 163.04 and 119.05 [M-H-pentose-COO]^−^. Compound 4 produced a [M-H]^−^ peak at* m*/*z* 577.14 and fragment ions at* m*/*z* 425.09, 407.08, 289.07, 245.08, 125.03, which are typical for dimeric procyanidins [24]. Fragmentation of dimeric procyanidins through retro-Diels-Alder (RDA) fission results in a fragment ion at* m*/*z* 425.09. The fragment ion at* m*/*z* 407.08 arises from subsequent elimination of water from the ion at* m*/*z* 425.09 [24, 25]. Heterocyclic ring fission (HRF) of the flavan-3-ol structure results in the loss of a phloroglucinol unit (*m*/*z* 125.03). Direct cleavage of the interflavanoid bonds results in the fragment ion at* m*/*z* 289.07 [24, 25]. Similarly, compound 6 was identified as trimeric procyanidins from its molecular ion [M-H]^−^ at* m*/*z* 865.21 and fragment ions at* m*/*z* 407.08, 289.07, 125.03 [25]. Compounds 7 and 10 were identified based on their molecular and fragment ions as previously described [24]. The identification of the flavonoid glycosides of* B. hookeri* was achieved based on the PDA and MS/MS data as previously described [23, 26, 27]. The MS/MS analysis of compound 9 revealed fragment ion peaks at* m*/*z* 301.04 and 300.03, which indicated the glycosylation site at the 3-position of quercetin [27]. Moreover, the MS/MS analysis of compound 9 indicated that no distinctive intermediate fragments: [M-H-146]^−^ and [M-H-162]^−^ were observed, confirming the rutinoside (rhamnosyl (1→6) glucoside) interglycosidic linkage [26]. Similarly, compounds 8, 11–13 were identified from the [M-H]^−^ and fragment ion peaks as well as from the retention time and distinctive PDA data.

### 3.2. Acute Toxicity

No adverse behavioral changes, toxicity symptoms, or mortality were observed in mice at doses up to 5000 mg/kg (LD_50_ > 5000 mg/kg). Based on these findings, BHE is considered safe in mice.

### 3.3. Hepatoprotective Effect

A significant increase in the activity of the serum enzymes ALT, AST, and ALP (*P* < 0.001) was observed in the CCl_4_ group compared to the negative control group (Table 2). The treatment of intoxicated mice with BHE at the tested doses (500 and 1000 mg/kg/day) produced a significant hepatoprotective effect and reduced the activity of ALT, AST, and ALP. Notably, BHE treatment at a dose of 1000 mg/kg was more effective in reducing the ALT, AST, and ALP levels compared to silymarin (Table 2). The levels of the hepatic enzymes were comparable and not significantly different from the normal control group.

### 3.4. Antioxidant Activity

**Figure 2:**
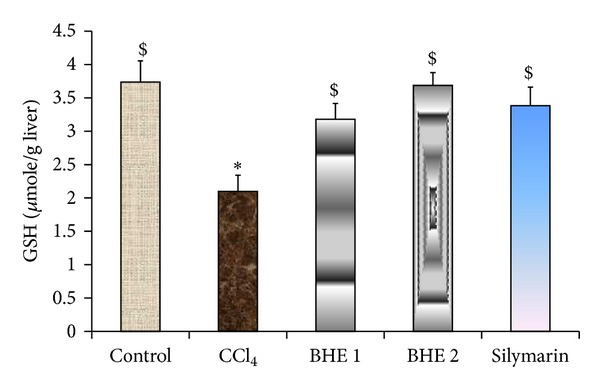
Effect of BHE and silymarin on the hepatic GSH level. Data are expressed as means ± SEM, (*n* = 9). BHE 1: CCl_4_ + 500 mg/kg of BHE. BHE 2: CCl_4_ + 1000 mg/kg of BHE. Values having different superscripts within the same column are significantly different at *P* < 0.05.

**Figure 3:**
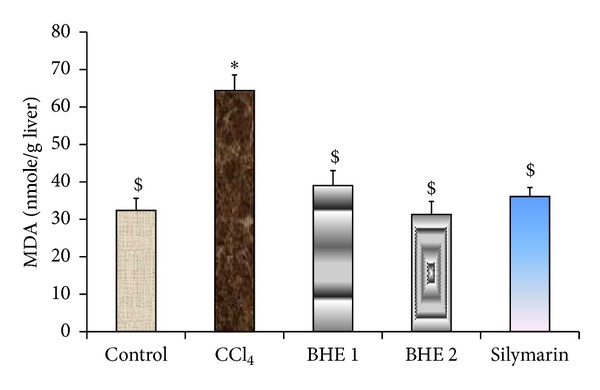
Effect of BHE and silymarin on lipid peroxidation (hepatic MDA level). Data are expressed as means ± SEM, (*n* = 9). BHE 1: CCl_4_ + 500 mg/kg of BHE. BHE 2: CCl_4_ + 1000 mg/kg of BHE. Values having different superscripts within the same column are significantly different at *P* < 0.05.

A marked reduction in hepatic GSH and antioxidant enzymes (GPx, GR, GST, and SOD) was observed in CCl_4_-intoxicated mice. In contrast, a significant increase in the MDA level was evident compared to the normal control group (Table 3). Administration of BHE at the two treatment doses (500 and 1000 mg/kg/day) produced a marked increase in GSH (by 51 and 76%, resp.) and improved the activity of all the antioxidant enzymes relative to the CCl_4_-intoxicated group (Table 3 and Figure 2). In addition, the elevated hepatic level of MDA was reduced by 39 and 51% at the tested doses, respectively, compared to the CCl_4_-intoxicated group (Figure 3). The higher dose of BHE was more effective in reducing the MDA level compared to silymarin. Notably, the GSH, GPx, GR, GST, and SOD levels in the group treated with 1000 mg/kg of BHE were markedly higher compared to the silymarin-treated group. Moreover, BHE treatment at a dose of 500 mg/kg induced a marked increase in the GR and GST levels compared to the silymarin-treated group. These results clearly indicated the strong* in vivo* antioxidant activity provided by BHE.

### 3.5. Histopathological Observations

**Figure 4:**
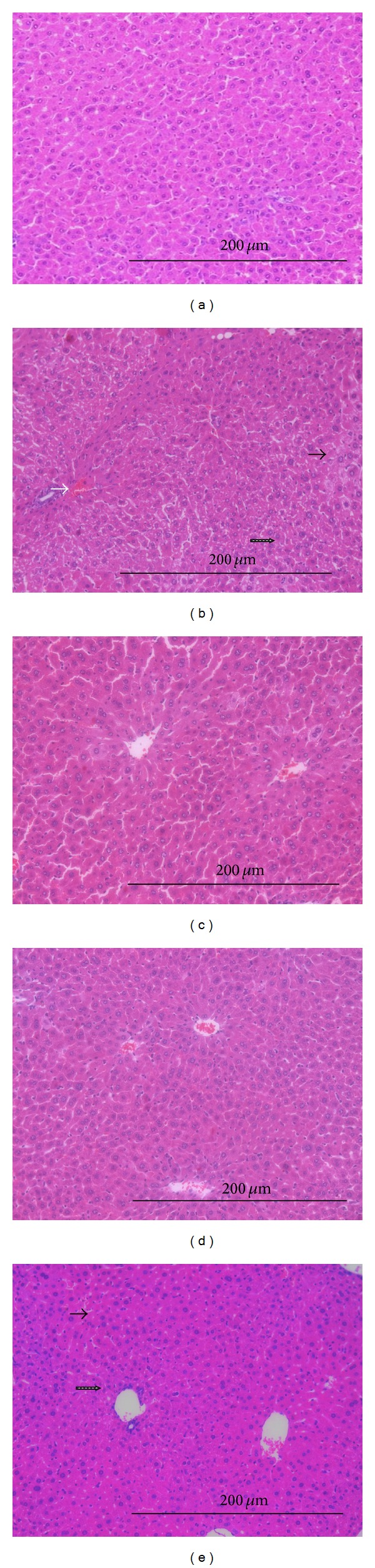
Hepatoprotective effect of BHE in CCl_4_-intoxicated mice. (a) Group I (normal control): showing normal hepatic architecture and normal hepatocytes. (b) Group II (CCl_4_-treated group): showing marked loss of hepatic architecture, marked hydropic degeneration, central vein congestion, and scattered lymphocytes in between the hepatocytes and in the sinusoids. (c and d) Groups III and IV (CCl_4_ + 500 mg/kg or CCl_4_ + 1000 mg/kg, respectively of BHE): showing preserved hepatic architecture. (e) Group V (CCl_4_ + 500 mg/kg of silymarin): showing normal hepatic architecture and mild hydropic degeneration of hepatocytes (H & E, 200x). Hydropic degeneration (the black arrow), central vein congestion (the white arrow), and scattered lymphocytes in between the hepatocytes and in the sinusoids (the dotted black arrow).

CCl_4_ induced severe loss of the hepatic architecture, marked hydropic degeneration, fatty changes, central vein congestion, Kupffer cell hyperplasia, necrosis, and scattered lymphocytes in between the hepatocytes and in the sinusoids. The pathological changes induced by CCl_4_ were markedly ameliorated in the groups treated with BHE at the two treatment doses (Figure 4 and Supplementary Data available online at http://dx.doi.org/10.1155/2014/245171). The liver architecture is preserved in 75 and 87.5% of animals treated with 500 and 1000 mg/kg of BHE, respectively. In addition, BHE conferred protection against liver damage as evidenced by the marked decrease in lymphocyte infiltration, hydropic degeneration, fatty changes, central vein congestion, Kupffer cell hyperplasia, and necrotic changes. It was clear that the lower dose of BHE is as effective as silymarin in reducing the Kupffer cell hyperplasia induced by CCl_4_ intoxication. Notably, treatment with BHE at doses of 500 and 1000 mg/kg reduced the fatty changes, central vein congestion, and necrosis more than in the silymarin-treated group. Moreover, the higher dose of BHE was more effective in restoring the hepatic architecture and in reducing the lymphocyte infiltration, Kupffer cell hyperplasia, and hydropic degeneration compared to the silymarin-treated group. Complete amelioration of the fatty changes was evident by the higher dose of BHE.

## 4. Discussion

CCl_4_ intoxication is a widely used experimental model for liver injury. The highly hepatotoxic metabolites, namely, trichloromethyl radicals (CCl_3_^·^ and CCl_3_O_2_^·^) are generated during the metabolic activation of CCl_4_ by cytochrome P-450. These radicals have a central role in the initiation of lipid peroxidation, inflammation, and fatty changes of the liver [3, 28]. Moreover, CCl_4 _intoxication is associated with oxidative stress since the CCl_3_^·^ and CCl_3_O_2_^·^ radicals alter the antioxidant state of the liver by deactivating the hepatic antioxidant enzymes including SOD, GPx, GR, and GST [3]. Trichloromethyl radicals also react with the sulfhydryl groups of GSH leading to its deactivation [3]. In the present study, CCl_4_ treatment markedly increased the levels of AST, ALT, and ALP. The leakage of the marker enzymes into the blood was associated with marked necrosis, loss of hepatic architecture, hydropic degeneration, fatty changes, Kupffer cell hyperplasia, central vein congestion, and infiltration of the liver by lymphocytes. The MDA level in the liver tissue was markedly increased in response to CCl_4_ intoxication, indicating oxidative damage of the liver. CCl_4_ administration also reduced the levels of GPx, SOD, GST, GSH, and GR in the liver tissue compared to the normal mice. The results of the present study demonstrated that treatment with BHE returned the increased MDA to its normal level. The inhibitory effect against lipid peroxidation suggested that BHE could prevent the liver injury induced by free radicals along with the subsequent pathological changes in the liver. The marked reduction in the leakage of liver enzymes into the serum also confirmed the inhibitory effect of BHE against lipid peroxidation. In contrast, the GSH, GPx, SOD, GST, and GR levels were markedly improved compared to the silymarin-treated group. Modulation of these antioxidant defenses clearly contributed to the antioxidant and hepatoprotective activity of BHE. In this study, the phytochemical composition of* B. hookeri* was identified for the first time using the HPLC-PDA-ESI/MS/MS analysis. The identified compounds include gallic acid, hydroxycinnamic acid derivatives, flavonoids, epigallocatechin gallate, and dimeric and trimeric procyanidins. The remarkable hepatoprotective and antioxidant effect of BHE may be attributed to a synergistic effect between these compounds [8]. Experimental evidence proved that the whole plant extracts usually possess much better pharmacological activities than single isolated ingredients due to synergistic interactions between the individual components [6, 7]. It is also known that mixtures of antioxidant compounds are more active than the individual components of these mixtures [29]. Flavonoids, especially flavonols and flavan-3-ols, possess various biological effects that contribute to health benefits including antioxidant and hepatoprotective activities [8, 29]. Flavonols prevent the oxidative stress by direct scavenging of free radicals, metal chelation, reduction of tocopheryl radicals, and induction of antioxidant enzymes as well as phase II detoxifying enzymes such as GST. Flavonoids also have a membrane-stabilizing effect [8, 9, 29]. Diverse pharmacological activities have been attributed to epigallocatechin gallate, including potent antioxidant, antifibrogenic, and hepatoprotective activity [8, 30]. Gallic acid has been reported to exhibit a strong antioxidant and hepatoprotective activity [31]. Proanthocyanidins are complex polymers of polyhydroxy flavan-3-ol constitutive units and are widely present in legumes and fruits [8]. Commercial preparations of standardized proanthocyanidins, including grape seed proanthocyanidin extract, are marked as dietary supplements due to their health benefits [32]. Proanthocyanidins are known to increase the activity of GST and SOD, to elevate the cellular GSH content, and to have potent radical-scavenging activity [8]. They also have an ameliorative effect on oxidative stress and hepatic fibrosis [32]. Grape seed proanthocyanidin extract exhibited a strong hepatoprotective as well as antifibrogenic effects against dimethylnitrosamine and thioacetamide induced liver injury in animal models [32, 33]. Sufficient evidence indicates that hepatocellular damage and subsequent infiltration by inflammatory cells have a central role in the activation of Kupffer cells to release several cytokines and free radicals, which in turn stimulate the transformation of hepatic stellate cells to myofibroblast-like cells, ultimately leading to the enhancement of collagen formation and hepatic fibrosis [32, 33]. The protective effect of BHE against Kupffer cell hyperplasia, infiltration by inflammatory cells, and other CCl_4_-induced pathological changes in liver, along with the protective effect against oxidative stress, indicated that this plant has hepatoprotective and antifibrotic therapeutic potential.

## 5. Conclusion

Based on the results of this study, the hepatoprotective effect of BHE is attributed to its ability to reduce the rate of lipid peroxidation, to enhance the antioxidant defense status, and to guard against the pathological changes of the liver induced by CCl_4_ intoxication. The strong* in vivo* antioxidant activity also suggests that a dietary supplement of BHE may confer a beneficial effect against oxidative stress. The hepatoprotective activity of BHE is concluded to be partly mediated by the antioxidant effect of its constituents. This study represents the first report that employed the HPLC-PDA-ESI/MS/MS technique for the identification of the phytoconstituents of* B. hookeri*. The adopted HPLC-PDA-ESI/MS/MS method provided a useful tool to develop a characteristic chromatographic fingerprint for the authentication of BHE and for the identification of its composition.

## Supplementary Material

Bauhinia hookeri ethanol extract (BHE) confered marked amelioration of the pathological changes induced by CCl4 at the two treatment doses (500 and 1000 mg/kg) as evidenced by the preserved hepatic architecture, the marked decrease in lymphocyte infiltration, hydropic degeneration, fatty changes, central vein congestion, Kupffer cell hyperplasia and necrotic changes. It was clear that the lower dose of BHE is as effective as silymarin in reducing the Kupffer cell hyperplasia induced by CCl4 intoxication. BHE treatment inhibited the fatty changes, central vein congestion and necrosis more than in the silymarin-treated group. Complete amelioration of the fatty changes was evident by the higher dose of BHE.

## Figures and Tables

**Table 1:** LC-PDA-ESI/MS/MS identification of the major constituents of *B. hookeri*.

*N*	*t*_*R*_ (min)	UV maximum (nm)	(M–H)^−^ *m*/*z*	Fragments (MS/MS) *m*/*z*	Tentative structural assignment
1	4.7	222, 271	169.01	125.02	Gallic acid
2	13.0	220, 225 (sh), 280, 320	311.04	179.04, 149.01, 135.05	Caffeic acid pentose ester
3	14.1	218, 230 (sh), 280, 325	311.04	179.04, 149.01, 135.05	Caffeic acid pentose ester
4	15.1	205, 225 (sh), 280	577.14	425.09, 407.08, 289.07, 245.08, 125.03	Procyanidin dimer (epi/catechin dimer)
5	16.4	205, 230 (sh), 280 (sh), 315 (sh)	295.05	163.04, 149.01, 119.05	Coumaric acid pentose ester
6	17.4	205, 225 (sh), 280	865.21	407.08, 289.07, 125.03	Procyanidin trimer (epi/catechin trimer)
7	17.7	220, 280	729.16	407.08, 289.07, 245.08, 169.02, 125.03	Procyanidin dimer gallate
8	18.2	200, 260, 355	615.11	463.09, 301.04, 300.03, 271.03, 255.03, 179.00, 151.01, 169.02, 125.03	Quercetin 3-O-hexoside gallate
9	18.5	210, 250, 356	609.16	301.04, 300.03, 271.02, 255.03, 179.00, 151.01	Rutin: Quercetin 3-O-rhamnosyl (1→6) hexoside
10	19.4	210, 278	441.09	289.07, 245.08, 169.02, 125.03	Epicatechin gallate
11	19.6	200, 260, 355	463.09	301.04, 300.03, 271.02, 255.03, 179.00, 151.00	Quercetin 3-O-hexoside
12	21.1	200, 265, 345	599.11	447.10, 285.04, 284.03, 255.03, 227.04, 169.02, 125.03	Kaempferol 3-O-galloyl hexoside
13	22.0	200, 265, 345	447.10	285.04, 284.03, 255.03, 227.04	Kaempferol 3-O-hexoside
14	28.4	220, 290 (sh), 318	441.09	295.05, 277.04, 163.04, 145.03, 119.05	Dicoumaroyl pentose
15	29.0	220, 290 (sh), 315	441.09	295.05, 277.04, 163.04, 145.03, 119.05	Dicoumaroyl pentose

**Table 2:** Effect of BHE on hepatic marker enzymes after 6 weeks of CCl_4_ intoxication in mice.

Animal groups	ALT (U/L)	AST (U/L)	ALP (IU/L)
Control	13.23 ± 0.71^$^	122.40 ± 2.68^$^	81.06 ± 4.73^$^
CCl_4_	43.25 ± 3.05*	223.30 ± 5.32*	143.65 ± 3.82*
BHE (500 mg/kg/day)	24.29 ± 2.60^#^ (44%)	142.84 ± 5.45^#^ (36%)	104.14 ± 5.44^#^ (28%)
BHE (1000 mg/kg/day)	15.76 ± 2.72^$#^ (64%)	120.24 ± 5.83^$^ (46%)	83.76 ± 5.86^$#^ (42%)
Silymarin (500 mg/kg/day)	23.64 ± 2.25^#^ (45%)	125.06 ± 3.43^$#^ (44%)	89.78 ± 5.24^$#^ (38%)

Data are expressed as the means ± SEM, (*n* = 9).

The numbers in parentheses represent the percentage of reduction from the CCl_4_-intoxicated group.

Values having different superscripts within the same column are significantly different at *P* < 0.05.

**Table 3:** Effect of BHE on lipid peroxidation (MDA), GSH, and antioxidant enzymes after 6 weeks of CCl_4 _intoxication in mice.

Animal groups	GSH (*μ*mole/g liver)	GPx (*μ*mole/min/g liver)	GR (*μ*mole/min/g liver)	GST (*μ*mole/min/g liver)	SOD (*μ*mole/min/g liver)	MDA (nmole/g liver)
Control	3.74 ± 0.32^$^	3.08 ± 0.29^$^	2.46 ± 0.23^$^	48.96 ± 4.87^$^	355.85 ± 7.26^$^	32.36 ± 3.26^$^
CCl_4_	2.10 ± 0.24*	1.64 ± 0.23*	1.57 ± 0.27^$^	20.85 ± 2.73*	236.56 ± 4.08*	64.37 ± 4.18*
BHE (500 mg/kg/day)	3.18 ± 0.24^$^ (+51%)	2.51 ± 0.24^$∗^ (+53%)	2.30 ± 0.27^$^ (+46%)	35.09 ± 2.47^$∗^ (+68%)	297.48 ± 4.86^#^ (+26%)	39.03 ± 3.97^$^ (−39%)
BHE (1000 mg/kg/day)	3.69 ± 0.19^$^ (+76%)	3.00 ± 0.26^$^ (+83%)	2.37 ± 0.33^$^ (+51%)	41.91 ± 4.31^$^ (+101%)	324.67 ± 6.28^§^ (+37%)	31.32 ± 3.47^$^ (−51%)
Silymarin (500 mg/kg/day)	3.38 ± 0.28^$^ (+61%)	2.51 ± 0.30^$∗^ (+53%)	2.19 ± 0.36^$^ (+39%)	33.58 ± 4.21^$∗^ (+61%)	315.32 ± 4.70^#§^ (+33%)	36.10 ± 2.39^$^ (−44%)

Data are expressed as the means ± SEM, (*n* = 9).

The numbers in parentheses represent the percent change from the CCl_4_-intoxicated group.

Values having different superscripts within the same column are significantly different at *P* < 0.05.
