# Supplementary material for: Hepatoprotective and Antioxidant Effect of Bauhinia hookeri Extract against Carbon Tetrachloride-Induced Hepatotoxicity in Mice and Characterization of Its Bioactive Compounds by HPLC-PDA-ESI-MS/MS
Source: Biomed Res Int. 2014 May 14;2014:245171. doi: 10.1155/2014/245171 (PMC4053259; doi:10.1155/2014/245171)
Supplement: Supplementary file 1 — Bauhinia hookeri ethanol extract (BHE) confered marked amelioration of the pathological changes induced by CCl4 at the two treatment doses (500 and 1000 mg/kg) as evidenced by the preserved hepatic architecture, the marked decrease in lymphocyte infiltration, hydropic degeneration, fatty changes, central vein congestion, Kupffer cell hyperplasia and necrotic changes. It was clear that the lower dose of BHE is as effective as silymarin in reducing the Kupffer cell hyperplasia induced by CCl4 intoxication. BHE treatment inhibited the fatty changes, central vein congestion and necrosis more than in the silymarin-treated group. Complete amelioration of the fatty changes was evident by the higher dose of BHE. [file 245171.f1.docx]

**Short Discription of the Supplementary Data for the Manuscript 245171.v2**

*Bauhinia hookeri* ethanol extract (BHE) confered marked amelioration of the pathological changes induced by CCl_4_ at the two treatment doses (500 and 1000 mg/kg) as evidenced by the preserved hepatic architecture, the marked decrease in lymphocyte infiltration, hydropic degeneration, fatty changes, central vein congestion, Kupffer cell hyperplasia and necrotic changes. It was clear that the lower dose of BHE is as effective as silymarin in reducing the Kupffer cell hyperplasia induced by CCl_4_ intoxication. BHE treatment inhibited the fatty changes, central vein congestion and necrosis more than in the silymarin-treated group. Complete amelioration of the fatty changes was evident by the higher dose of BHE.

Effect of BHE on histopathological changes after 6 weeks of CCl_4_ intoxication in mice

| Groups | Hepatic architecture | | | Hydropic degeneration | | | | Fatty changes | | Central vein congestion | | Kupffer cell hyperplasia | | Necrosis | | Infiltration of portal tracts by lymphocytes | |
| --- | --- | --- | --- | --- | --- | --- | --- | --- | --- | --- | --- | --- | --- | --- | --- | --- | --- |
|  | Preserved | Partial loss | Lost | Marked | Moderate | Mild | Absent | Present | Absent | Present | Absent | Present | Absent | Present | Absent | Present | Absent |
| Normal Control | 100% | 0 | 0 | 0 | 0 | 0 | 100% | 0 | 100% | 0 | 100% | 0 | 100% | 0 | 100% | 0 | 100% |
| CCl_4_ | 0 | 37.5% | 62.5% | 62.5% | 37.5% | 0 | 0 | 75% | 25% | 62.5% | 37.5% | 75% | 25% | 100% | 0 | 100% | 0 |
| BHE (500mg/kg/day) | 75% | 25% | 0 | 12.5% | 25% | 50% | 12.5% | 12.5% | 87.5% | 37.5% | 62.5% | 37.5% | 62.5% | 25% | 75% | 37.5% | 62.5% |
| BHE (1000mg/kg/day) | 87.5% | 12.5% | 0 | 0 | 0 | 37.5% | 62.5% | 0 | 100% | 25% | 75% | 25% | 75% | 25% | 75% | 12.5% | 87.5% |
| Silymarin (500mg/kg/day) | 75% | 25% | 0 | 0 | 0 | 50% | 50% | 37.5% | 62.5% | 50% | 50% | 37.5% | 62.5% | 37.5% | 62.5% | 25% | 75% |

Data are presented as the percentage of the histopathological changes observed in the total number of animals examined (n=8).

The parameters examined with their relative score systems for the assessment of histological damage were as follows; hepatic architecture: preserved, partial loss or complete loss; hydropic degeneration: absent, mild, moderate or marked; fatty changes: absent or present; central vein congestion: present or absent; Kupffer cell hyperplasia: absent or present; necrosis: absent or present and infiltration of portal tract by lymphocytes: absent or present.
